# Four Dimensional Digital Tomosynthesis Using on-Board Imager for the Verification of Respiratory Motion

**DOI:** 10.1371/journal.pone.0115795

**Published:** 2014-12-26

**Authors:** Justin C. Park, Jin Sung Kim, Sung Ho Park, Matthew J. Webster, Soyoung Lee, William Y. Song, Youngyih Han

**Affiliations:** 1 Department of Radiation Oncology, University of Florida, Gainesville, Florida, United States of America; 2 Department of Radiation Oncology, Samsung Medical Center, Sungkyunkwan University School of Medicine, Seoul, Korea; 3 Department of Neurosurgery, Ulsan University Hospital, Ulsan, Korea; 4 Department of Radiation Oncology, University of Texas Southwestern Medical Center, Dallas, United States of America; 5 Department of Radiation Oncology & Medical Biophysics, Sunnybrook Health Sciences Centre, University of Toronto, Toronto, Canada; University of Nebraska Medical Center, United States of America

## Abstract

**Purpose:**

To evaluate respiratory motion of a patient by generating four-dimensional digital tomosynthesis (4D DTS), extracting respiratory signal from patients' on-board projection data, and ensuring the feasibility of 4D DTS as a localization tool for the targets which have respiratory movement.

**Methods and Materials:**

Four patients with lung and liver cancer were included to verify the feasibility of 4D-DTS with an on-board imager. CBCT acquisition (650–670 projections) was used to reconstruct 4D DTS images and the breath signal of the patients was generated by extracting the motion of diaphragm during data acquisition. Based on the extracted signal, the projection data was divided into four phases: peak-exhale phase, mid-inhale phase, peak-inhale phase, and mid-exhale phase. The binned projection data was then used to generate 4D DTS, where the total scan angle was assigned as ±22.5° from rotation center, centered on 0° and 180° for coronal “half-fan” 4D DTS, and 90° and 270° for sagittal “half-fan” 4D DTS. The result was then compared with 4D CBCT which we have also generated with the same phase distribution.

**Results:**

The motion of the diaphragm was evident from the 4D DTS results for peak-exhale, mid-inhale, peak-inhale and mid-exhale phase assignment which was absent in 3D DTS. Compared to the result of 4D CBCT, the view aliasing effect due to arbitrary angle reconstruction was less severe. In addition, the severity of metal artifacts, the image distortion due to presence of metal, was less than that of the 4D CBCT results.

**Conclusion:**

We have implemented on-board 4D DTS on patients data to visualize the movement of anatomy due to respiratory motion. The results indicate that 4D-DTS could be a promising alternative to 4D CBCT for acquiring the respiratory motion of internal organs just prior to radiotherapy treatment.

## Introduction

During the past decades, there has been a significant growth in radiation therapy technologies for effective cancer treatment. The present state of imaging and radiation treatment technologies have enabled high conformal dose delivery to the target volume, and one of main contributions has come from the introduction of image-guided radiation therapy (IGRT).

One of the processes in IGRT is to verify the patient's position before the treatment. It provides the anatomical information of the patient prior to or at the time of the treatment to increase the precision of target localization in radiotherapy. In order to minimize the dose delivered to the surrounding tissue while escalating the dose delivered to the tumor, an accurate positioning of the patient before the treatment is necessary. Initially, two-dimensional (2D) orthogonal kilo-voltage (kV) imaging had been widely used to verify the patient position [1], [2]. The 2D orthogonal kV imaging is quick to implement, but it may contain considerable errors since anatomical information of the patients is insufficiently considered during the verification [3]. The advent of the cone beam computed tomography (CBCT) system with an on-board imager (OBI) has provided the capability of three-dimensional (3D) image guidance for verifying the positioning [4]–[7]. Consequently, the use of CBCT has made verification of patient positioning for treatment more accurate, compared to that of the conventional method. With the help of OBI, various studies regarding kV-CBCT and mega voltage CBCT (MV-CBCT) are being carried out in order to investigate and optimize the accuracy of patient positioning [8]–[12].

In IGRT, it is crucial to investigate the exact movement of tumors, which is still a challenging issue. Thoracoabdominal regions, such as parts of the lung and liver, move due to breathing, and this respiratory motion causes significant artifacts when using 3D CBCT in current OBI systems [13]–[15]. These effects may lead to inconsistencies in dose delivery and may increase toxicity to surrounding normal tissues during the treatment. In order to improve the motion artifact due to respiratory motion, respiratory correlated four-dimensional (4D) CBCT has been developed in various institutions and its clinical applications are beginning to be carried out [16]–[21].

The use of CBCT, however, requires a relatively long image acquisition time (∼1min) for a complete image dataset which is due to limitation of the full gantry rotation speed in LINACs and potential mechanical collisions for large or off-center patient setups. This leads to a relatively high imaging dose to the patient and therefore, daily localization of the patient for the duration of the fractionation schedule could be problematic [22], [23]. In addition, several reports have shown that for 4D-CBCT, from retrospective sorting and binning of projection data, based on respiratory phases, aliasing effects occurs due to the non-uniform limited number of projections over the full gantry rotation [24], [25].

Digital tomosynthesis (DTS) has been developed and used for many decades for diagnostic applications [26]–[29]. It is a fast, low-dose, alternative to CBCT where a stack of DTS volume can be reconstructed from single limited angle scan ranging from 10° to 40°. Although it does not have axial information such as in CT, it provides high quality information on the sagittal and coronal part of the anatomy. In recent years, there has been growing concerns to adapt DTS for image guidance for radiotherapy applications and several studies have reported that the DTS imaging on OBI has comparable positioning accuracy to CBCT [3], [30], [31]. Moreover, a recent experimental study on the on-board four-dimensional (4D) DTS has shown a great potential for respiratory correlated 4D imaging using OBI and it has suggested that motion artifacts were substantially less severe when compared with 4D-CBCT [32]. Based on the approaches mentioned above, we have implemented 4D-DTS on patient data. The purpose of this study is to verify the respiratory motion of the patient, and compare its quality with 4D-CBCT which we have also implemented.

## Methods and Materials

### 1. Patient imaging data

This study was approved by the Institutional Review Board (IRB) of the Samsung Medical Center (SMC 2014-08-100) and patient consents were specifically waived because the data were analyzed anonymously and approval was given by the IRB. Patient confidentiality and privacy were protected according to national standard. In this study, four clinical cases (2 lung and 2 liver), using fractionated stereotactic radiotherapy, were selected to illustrate the potential of 4D DTS for moving organs. Each patient was initially scanned for 3D image guidance, and their data sets were later sent for retrospective 4D CBCT and DTS reconstruction. The projection data of the selected patients was acquired using an on-board imager (OBI Version 1.4) CBCT system (Varian Medical Systems, Inc., Palo Alto, CA) which consists of a-Si flat panel detector and kV X-ray source mounted on a Varian 21EX Clinac. The imaging system is mounted orthogonal to the treatment system sharing approximately the same rotation center. The flat panel detector consists of 1024×768 pixels with a uniform 0.388×0.388 mm pixel size. The measured source to detector distance (SDD) was approximately 150 cm with a gantry rotation speed of 6 degrees per second which takes about 1 min for a full gantry rotation.

The CBCT images the two treatment sites were acquired in two different modes: “Low-dose thorax” mode for lung cancer patients and “Standard dose 150 cm bowtie” mode for liver cancer patients. Although the basic geometrical condition for all patients was “half-fan,” which is used to obtain a large field-of-view (FOV) for imaging large anatomical sites, imaging conditions were slightly different. In the case of the lung cancer patients, where “Low-dose thorax” mode was used, a total of 660 projections over 364° were acquired with a technique of 110 kVp, 20 mA and 20 ms for each projection. For the “Standard dose 150 cm bowtie” mode, 651 projections were acquired over 364° with 120 kVp, 80 mA and 25 ms for each projection. The FOV of both cases was 50×50 cm. Both projection techniques were acquired with an aluminum bowtie filter placed directly under X-ray tube to compensate the large area projection geometry.

### 2. Extraction of respiratory signal and phase assignment

**Figure 1:**
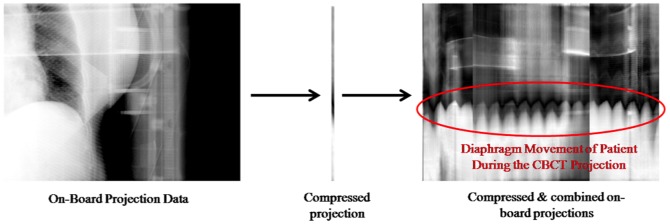
Scheme of generating the motion of the diaphragm (Amsterdam shroud) through compressing and combining 2D projection data.

**Figure 2:**
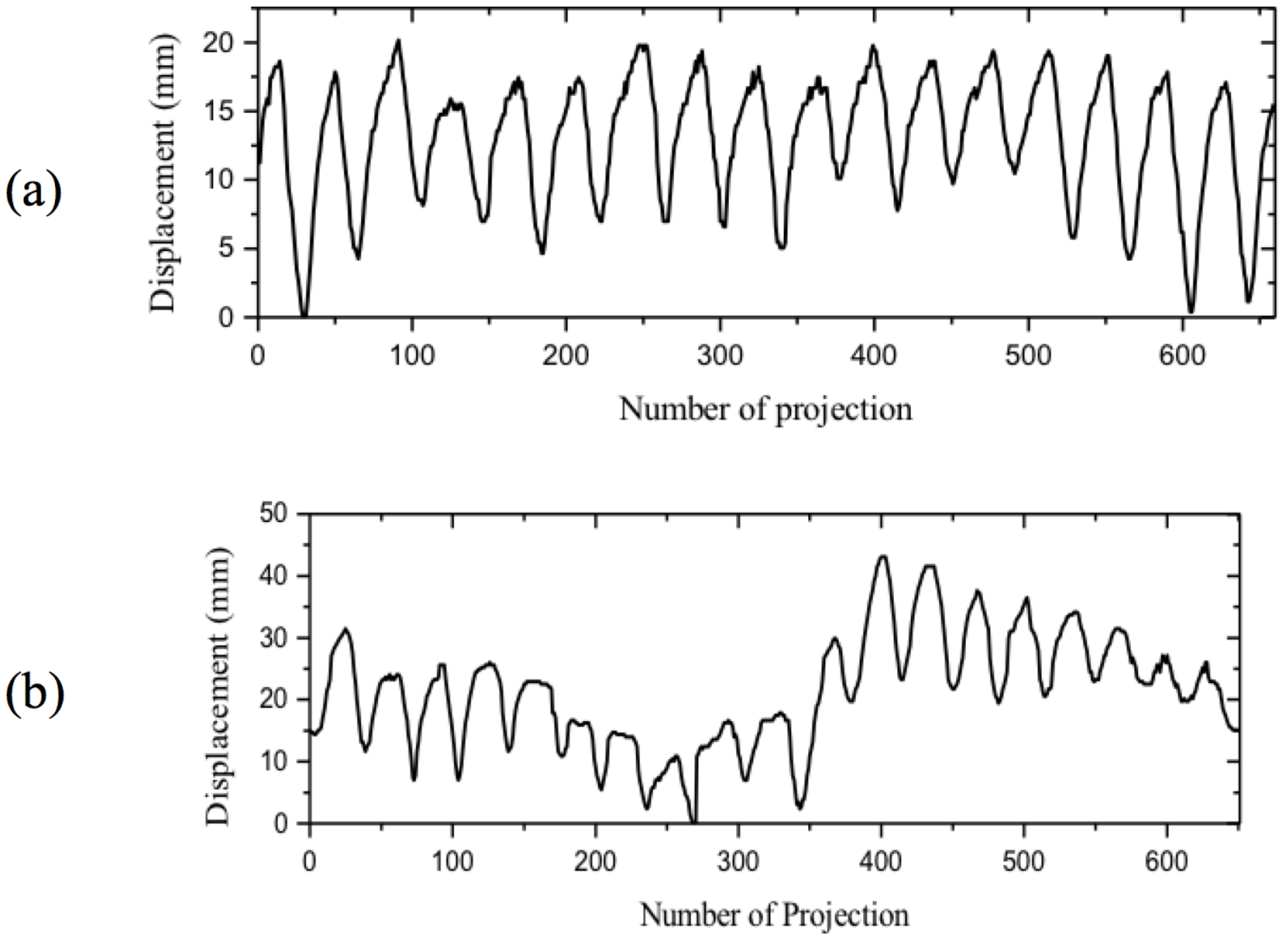
Extracted respiratory signals of two patients of lung (a) and liver (b) cases.

4D imaging requires a respiratory signal in order to assign a phase and sort the acquired projection data accordingly. In this study, we have imported an algorithm using diaphragm information which was proposed by Sonke et al [19]. It is a method which extracts a breathing signal that directly corresponds to the motion of the diaphragm (Amsterdam shroud) from acquired projection data automatically. It is based on the fact that influences of frame by frame changes of projection data on the lateral direction due to respiratory motion are considerably higher than changes due to gantry rotation. The Amsterdam shroud can be represented by compressing the 2D-projected image into 1D through the cranio-caudal axis, and combining all projections into 2D. Fig. 1 shows the scheme of generating the Amsterdam shroud through compressing and combining 2D projection data. The Amsterdam shroud during full gantry rotation can then be extracted as signal through an image processing scheme such as a Hilbert transformation or an adaptive thresholding technique [19]. Fig. 2 represents the extracted respiratory signals of two patients which we have included in this study.

**Figure 3:**
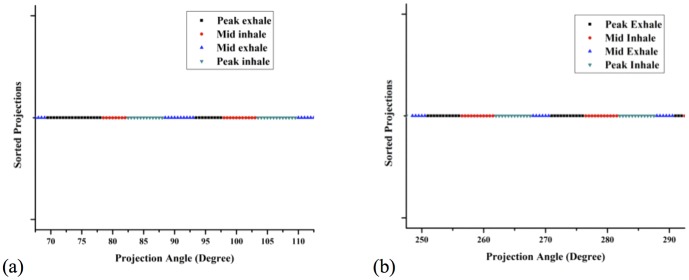
Plot of Lung Patient #1 patient's case where projections are sorted to each four phase bins with respect to corresponding projection angle.

The extracted signal from the stack of compressed projection data was then analyzed to determine the phase. Through assigning the minimum and maximum points after each cycle we have divided the projection data into four phases: peak-exhale phase, mid-inhale phase, peak-inhale phase, and mid-exhale phase. The reason for assigning fewer phases than other 4D-related studies is due to the insufficient amount of projection data due to restrictions in our hospital as specified earlier. Fig. 3 represents an example of a patient's case (Lung Patient #1) where projections are sorted to each of the four phase bins with respect to corresponding projection angle. It shows that the angular range assigned to each of the four phases is over 5 degrees per phase per breathing cycle (>10 projections/phase) which is tight but yet sufficient to reconstruct each volume without severe under-sampling. To sort projection data retrospectively, we have set minimum and maximum amplitudes of extracted signal for each period as 0% and 50% phase. The range of each phase was assigned according to the distance range of a single respiratory period between the minima and maxima measured. In this paper, −87.5% to 12.5% was assigned as the peak-exhale phase, 12.5% to 37.5% as the mid-inhale phase, 37.5% to 62.5% as the peak-inhale phase and lastly, 62.5% to 87.5% was assigned as the mid-exhale phase, respectively.

### 3. On-board 4D CBCT reconstruction

The 4D CBCT reconstruction resembles the approach of reconstructing dynamic volumetric cardiac imaging which was proposed by Wang et al [33]. Since each cone beam projection represents a snapshot of a certain respiratory phase, a set of projections can represent a set of different phases. According to the phase which we have assigned above, projection data can be sorted into several phases which can be fed into a separate reconstruction algorithm to generate the 4D datasets.

The reconstruction was accomplished with the well known FeldKamp (FDK) algorithm, which performs backprojection after filtration of the projection data to reconstruct each sorted phase [34]. The FDK algorithm was modified in order to suit the “half-fan” mode of cone-beam projection geometry. The resolution of the voxel grid was set to 512×512×64 with a resolution of approximately 1.0 mm (LR) ×1.0 mm(AP) ×2.5 mm(CC).

### 4. On-board 4D DTS reconstruction

On-board 3D DTS using an FDK-type algorithm is reconstructed from the subsets of projection data which was acquired for 3D CBCT since the present OBI system could not support the imaging sequence of DTS [30]-[32], [35], [36]. This can be also applied to reconstruct 4D DTS from subsets of 4D projection datasets for reconstructing 4D CBCT since the both DTS and CBCT share the same imaging conditions of the patient. The theory of reconstructing DTS is almost the same as CBCT, except that DTS is created under limited angle projections where as CBCT is reconstructed over all angle projections.

**Figure 4:**
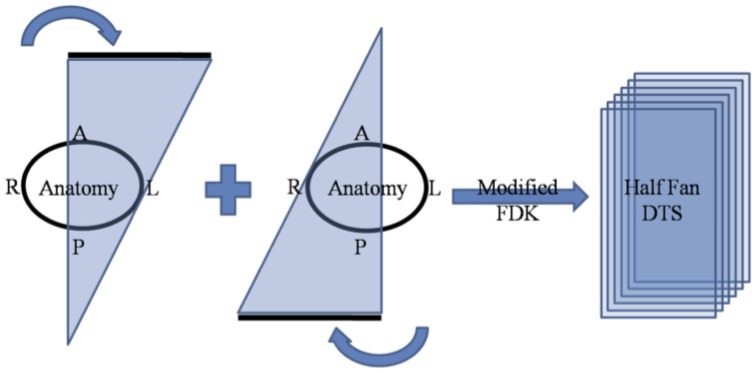
Diagram of acquiring projection data to generate coronal “half-fan” DTS.

In this study, we have reconstructed DTS in the coronal view where the X-ray source rotation is centered on 0° and 180°. The reason for using two projection scans from opposite directions was to visualize the full FOV of the patient which is insufficient when a single rotation center is used in the “half-fan” projection geometry. Fig. 4 shows how the coronal DTS is generated in the “half-fan” condition. Similarly, the sagittal view of DTS can be generated by using X-ray source rotations centered on 90° and 270°. For reconstructing 4D DTS, projection data which matched the above conditions from the 4D sorted bins were selectively used. The total scan angle was assigned as ±22.5° from the rotation center and 162 projections (81×2) were used for reconstruction of the DTS. In order to compare the 4D DTS result with the 4D CBCT result, the resolution of the voxels was set to be the same as in the 4D CBCT case.

## Results

### 1. Phantom Evaluation

For the evaluation of respiratory motion in our study, a QUASAR phantom with an embedded 2 cm diameter spherical object was scanned on a programmable motion platform with a sine wave function. The object motion period and amplitude were manually set to 4.0 seconds and 2 cm, respectively. With the acquired projection scans, we have reconstructed 4D CBCT and 4D DTS for all phases. The displacement of the object between the peak-exhale and peak-inhale phases was measured and compared at the central slice of the moving object.

**Figure 5:**
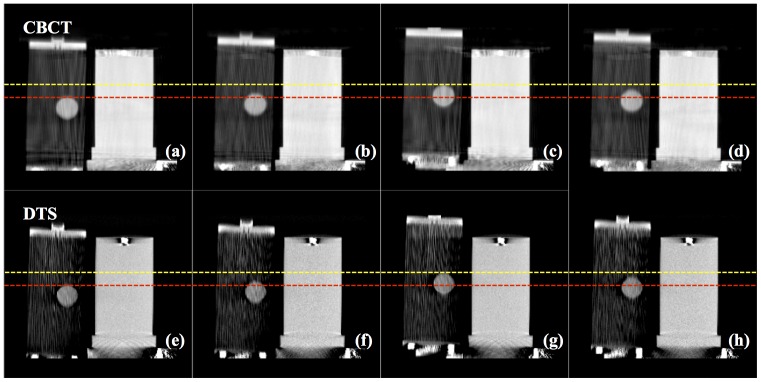
Coronal 4D CBCT and 4D DTS images of QUASAR phantom with peak-exhale (a), (e), mid-inhale (b), (f), peak-inhale (c), (g) and mid-exhale (d), (h) phases.

Fig. 5 displays coronal 4D CBCT and 4D DTS images of the QUASAR phantom at 0%-peak-exhale, 25%-mid-inhale, 50%-peak-inhale, and 75%-mid-exhale. It is evident that both 4D datasets reveal the phantom motion trajectory. The measured displacement from peak-to-peak phase was 1.98 and 2.01 cm for 4D CBCT and 4D DTS respectively. The discrepancy is 0.02 cm is well below the resolution limit of each voxel (0.25 cm).

### 2. 4D DTS of liver and lung cancer patients

**Figure 6:**
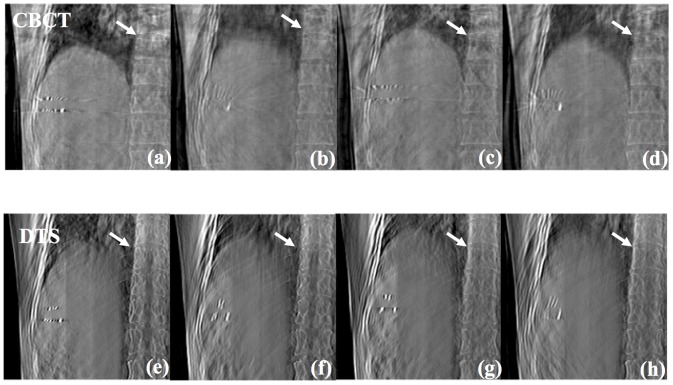
Sagittal and coronal DTS images reconstructed for the case of liver cancer patient #1 with 3D (a), peak-exhale (b), mid-inhale (c), peak-inhale (d), and mid-exhale (e) phases, emphasizing the patient's diaphragm and gold marker.

Fig. 6 shows coronal and sagittal 3D DTS images, (a,f) and 4D DTS images (b-e, g-j), with emphasis on the diaphragm and gold marker for the case of liver treatment reconstructed with conditions specified earlier. The motion of the diaphragm is apparent from the 4D reconstructed DTS images for the peak-exhale, mid-inhale, peak-inhale and mid-exhale phase assignments, which is absent in 3D DTS data. It is even clearer from the sagittal views, where the position of gold marker changes with the phase in 4D DTS data (white lines). Also, it is shown that the overall trajectory of gold marker at the 3D data is divided into the four states which represent the trajectory of motion during its respective phases. However, the blurring effect on the edge of diaphragm is much more severe on mid phases (c, e, h and i) than peak phases since the motion range on mid phases is comparably higher than the peak phases. In addition, the motion effect of the gold marker due to coverage of the higher motion range on the mid phases can be seen much more clearly. This problem can be improved by increasing the number of phases but would cause degradation to the image quality because the amount of projection data for reconstructing each of the phases would also decrease.

**Figure 7:**
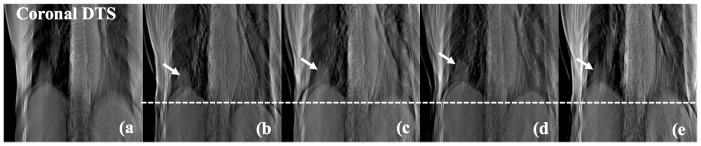
Coronal DTS images of lung cancer patient #1 emphasizing the cancer (white arrow) and diaphragm with 3D (a), peak-exhale (b), mid-inhale (c), peak-inhale (d) and mid-exhale (e) phases.

Fig. 7 represents a coronal 3D DTS image (a) and 4D DTS images (b–e) of a lung cancer patient. The ball-like shape which pointed to by the white arrow is the tumor volume. Referring to the figure, the motion of diaphragm and tumor according to the assigned phases is explicit.

### 3. 4D DTS and 4D CBCT comparison

**Figure 8:**
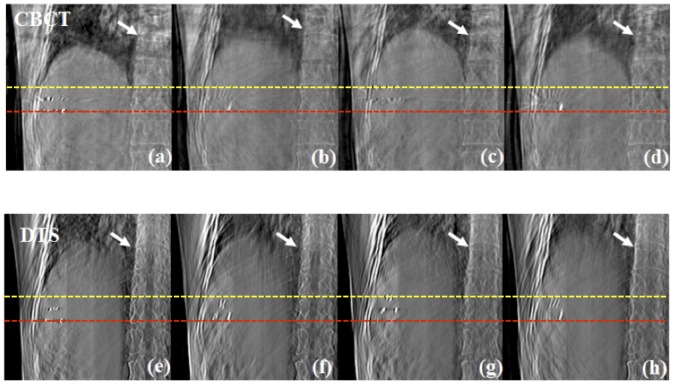
Comparative result (Coronal view) between 4D CBCT and DTS of liver cancer patient #1 case with peak-exhale (a), (e), mid-inhale (b), (f), peak-inhale (c), (g) and mid-exhale (d), (h) phases.

**Figure 9:**
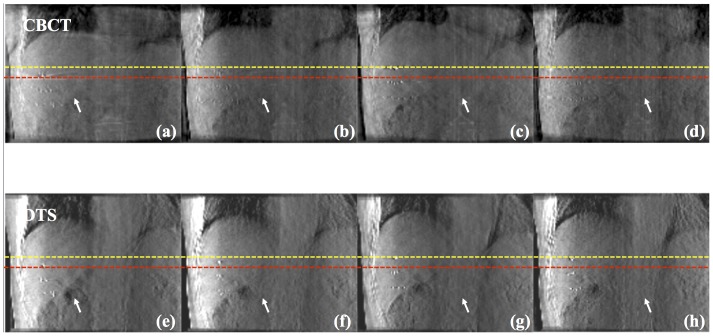
Results (Coronal view) showing 4D CBCT and DTS of liver cancer patient #2 with peak-exhale (a), (e), mid-inhale (b), (f), peak-inhale (c), (g) and mid-exhale (d), (h) phases.

Fig. 8 shows the comparative result between coronal images of the 4D CBCT and 4D DTS for liver cancer patients. Note that we have reconstructed both CBCT and DTS data with a single CBCT projection acquisition procedure. The anatomical information of bone seems to be distorted in the 4D CBCT images where as the boundary between bone and soft tissue is clearly distinguishable in 4D DTS images. It is primarily from the view aliasing effect, which is due to the loss of projection data at arbitrary angles through phase binning and sorting, which distorts the anatomical information in reconstruction space in CBCT images. The displacements of the markers between peak-exhale (red dashed line) and peak-inhale (yellow dashed line) phases were 3.21 cm and 3.16 cm for 4D CBCT and 4D DTS respectively. Fig. 9 displays coronal images of 4D CBCT and 4D DTS for the other liver cancer patient's case. It is seen that the boundary between liver and surrounding soft tissue (white arrow) is more clearly distinguishable in 4D DTS that 4D CBCT images. The marker displacements between peak-to-peak phases (yellow and red dashed lines) were measured to be 1.62 and 1.59 cm for 4D CBCT and 4D DTS.

**Figure 10:**
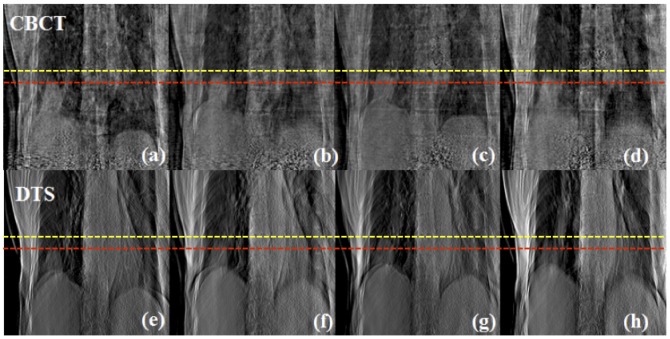
Coronal view of 4D CBCT and DTS reconstructed using lung cancer patient #1 scans with peak-exhale (a), (e), mid-inhale (b), (f), peak-inhale (c), (g) and mid-exhale (d), (h) phases.

**Figure 11:**
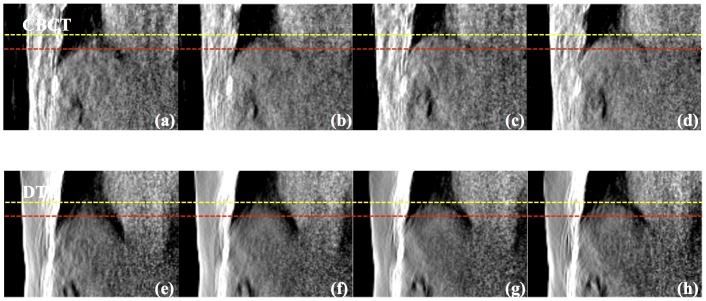
Comparative result (Coronal view) between 4D CBCT and DTS of lung cancer patient #2 case with peak-exhale (a), (e), mid-inhale (b), (f), peak-inhale (c), (g) and mid-exhale (d), (h) phases.

Fig. 10 shows the comparative result for one of the lung cancer patients at all phases. The displacements between peak-exhale (red dashed line) and peak-inhale (yellow dashed line) locations of tumor were measured to be 1.75 cm and 1.77 cm for 4D CBCT and 4D DTS. It can also be seen that the distortion effect in the patient's anatomy, due to limited angle projections and scatter effect from the use of a bowtie filter, is much more severe in the 4D CBCT images than the 4D DTS images. Fig. 11 displays coronal images of 4D CBCT and 4D DTS for the other lung case. The tumor displacements between the peak-to-peak phases (yellow and red dashed lines) for 4D CBCT and 4D DTS were measured to be 2.95 cm and 2.89 cm, respectively.

**Figure 12:**
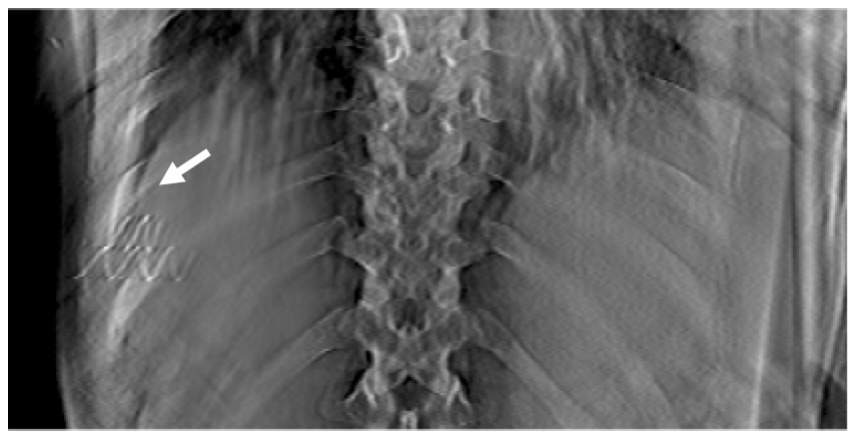
Residual blur of metal from outer plane cause significant amount of distortion of the patient's anatomy.

Fig. 12 shows the comparison between 4D CBCT and DTS of the gold marker implanted in the liver cancer patient at peak phases. In the CBCT images it can be seen that the anatomical information on the axial plane near the marker position is distorted due to a streak artifact generated from the presence of metal. In addition, when the markers are placed at the same slice plane, the anatomical information of the patient between the two metals is totally missing due to superposition of two metal streaks. On the other hand, we can hardly see these kinds of distortion effects at the slice plane of the 4D DTS images. This is due to the limited angle nature of DTS, since projections from the angle which is orthogonal to our point of view are not accounted for in the DTS reconstruction. 

## Discussion

In this study, we have shown the clinical feasibility of 4D DTS based on patients' data. In particular, the 4D DTS have been reconstructed using a respiratory signal extracted directly from 2D projection images from 3D CBCT acquisition. The phase assignment technique can be dependent upon enhancing the accuracy of 4D DTS; however, the phase based binning and sorting technique used in generating 4D CBCT/4D CT images have enabled the derivation of the real movement of the diaphragm even in 4D DTS images. The effects of irregularities based upon several factors such as amplitude, temporal phase distribution and frequency were not evaluated in this study. Therefore quantitative studies of such effects on DTS need to be evaluated and taken into account in the treatment planning process and positioning strategies.

We have generated 4D images of both CBCT and DTS with a single gantry rotation procedure. Since the number of acquired projections used in each phase were significantly limited, the resultant quality of the reconstructed images were too poor to be used in direct clinical application. Moreover, significant motion blur could be observed in mid-exhale and inhale phases since the coverage of the motion range on each phase were considerably higher due to a fewer phase assignments. This can be improved by increasing the number of projections or sampled data of the patient through increasing the frame rate of the imaging detectors and/or increasing the number of gantry rotations of the OBI. Such studies are being done in various other institutes for 4D CBCT imaging, but these kinds of studies focused on DTS imaging is also necessary for the enhancement of 4D DTS.

Radiation dose is one of the main concerns in particular for CBCT imaging. Although the present 4D study did not involve measurement or calculation of the imaging dose from 4D DTS scanning, the actual DTS imaging doses can be estimated using other studies of imaging doses. Since approximately one-fourth of the total CBCT projection acquisition (±22.5° from 90° and 270° in the case of sagittal DTS and ±22.5° from 0° and 180° in the case of coronal DTS) is necessary in generating volume data for the “half-fan geometry” DTS, it could be a promising alternative to CBCT imaging with respect to patient's imaging dose.

The quality of sagittal DTS was not sufficient for visualizing diaphragm movement in the case of the lung cancer patients. This is due to the substantial X-ray scatter and insufficient amount of detector signal which comes from the use of the low-dose imaging mode, bowtie filter and wider patient thickness through sagittal projection. This can be improved by increasing the current of the X-ray, scanning angle, or frame rate of the imaging detector. However, increasing the current of the X-ray or scanning angle will also increase radiation dose to the patient.

Reducing the blur residual artifact which comes from outer slice planes needs to be carried out to enhance the overall quality of the 3D as well as 4D DTS. Especially in the case of the patient with the gold marker, residual blur of the metal from the outer plan cause a significant amount of distortion in the patient's anatomy, as shown in Fig. 6a for the 3D DTS case (Fig. 12). Nevertheless, image distortion due to the presence of the metal is much less severe than that of 4D CBCT.

In this paper, we did not present the quantitative studies for the localization errors using 4D DTS, as this was not the intent of our study, but feel that it is appropriate to point out that three dimensional 4D reference DTS (RDTS) images are required for the verification process for planning 4D CT images with respiratory signals of patient synchronizing, a criterion for assigning the phase of respiratory motion with 4D DTS.

While tomosynthesis has been proposed for IGRT applications, most implementations have involved pretreatment acquisitions based on arc trajectories. Since a tomosynthesis acquisition typically requires the rotation of the slow-moving linac gantry through a range of 15°–60° of arc, the process of obtaining the tomosynthesis projection dataset is inconvenient for image guidance during the delivery of a treatment fraction. Recently, Maltz *et al.* described a new concept using a digital tomosynthesis method for patient setup and motion management in external beam radiation therapy [37]. With such DTS dedicated system, there is potential for 4D DTS to be used as powerful and convenient patient verification tool for IGRT in the future.

## Conclusion

We have implemented 4D DTS on patient data for the verification of respiratory motion. The method was based on direct extraction of respiratory signal from 2D projection images for 3D CBCT. Our study has shown that the discrepancy of peak-to-peak displacements of motion with 4DCBCT was less than 0.5 mm which was well below the resolution limit of each reconstructed voxel (2.5 mm). Such a discrepancy range showed good agreement with the related studies [38]. The results obtained from patient data have shown that the motion artifacts in such a 4D DTS dataset were substantially reduced compared to a 3D DTS scan. Moreover, 4D DTS has shown a better performance in terms of the view aliasing effect as well as visualization of metallic objects implanted in patient's anatomy compared to 4D CBCT, which we have also implemented. A verification process using 4D DTS will reduce respiration induced geometrical uncertainties, enabling safe delivery of 4D radiotherapy such as gated radiotherapy with small margins.

## References

[1] Herman MG (2005) Clinical use of electronic portal imaging. Semin. Radiat. Oncol. 15:157–167. 15983941 10.1016/j.semradonc.2005.01.002

[2] Byrne TE (2005) A review of prostate motion with considerations for the treatment of prostate cancer. Med. Dosim. 30:155–161. 16112467 10.1016/j.meddos.2005.03.005

[3] Yoo S, Wu QJ, Godfrey D, Yan H, Ren L, et al (2009) Clinical Evaluation of Positioning Verification Using Digital Tomosynthesis and Bony Anatomy and Soft Tissues for Prostate Image-Guided Radiotherapy. Int. J. Radiat. Oncol., Biol., Phys. 73:296–305. 19100923 10.1016/j.ijrobp.2008.09.006 PMC2665294

[4] Kupelian PA, Lee C, Langen KM, Zeidan OA, Mawn RR, et al (2008) Evaluation of image-guidance strategies in the treatment of localized prostate cancer. Int. J. Radiat. Oncol., Biol., Phys. 70:1151–1157. 17892920 10.1016/j.ijrobp.2007.07.2371

[5] Morin O, Gillis A, Chen J, Aubin M, Bucci MK, et al (2006) Megavoltage cone-beam CT: System description and clinical applications. Med. Dosim. 31:51–61. 16551529 10.1016/j.meddos.2005.12.009

[6] Oldham M, Letourneau D, Watt L, Hugo G, Yan D, et al (2005) Cone-beam-CT guided radiation therapy: A model for on-line application. Radiother and Oncol. 75:271–278. 10.1016/j.radonc.2005.03.026 15890419

[7] Park JC, Park SH, Kim JH, Yoon SM, Song SY, et al (2012) Liver motion during cone beam computed tomography guided stereotactic body radiation therapy. Med. Phys. 39:6431–6442. 23039678 10.1118/1.4754658

[8] Chang JW, Sillanpaa J, Ling CC, Seppi E, Yorke E, et al (2006) Integrating respiratory gating into a megavoltage cone-beam CT system. Med. Phys. 33:2354–2361. 16898437 10.1118/1.2207136

[9] Oelfke U, Tucking T, Nill S, Seeber A, Hesse B, et al (2006) Linac-integrated kV-cone beam CT: Technical features and first applications. Med. Dosim. 31:62–70. 16551530 10.1016/j.meddos.2005.12.008

[10] Pouliot J, Bani-Hashemi A, Chen J, Svatos M, Ghelmansarai F, et al (2005) Low-dose megavoltage cone-beam CT for radiation therapy. I Int. J. Radiat. Oncol., Biol., Phys. 61:552–560. 15736320 10.1016/j.ijrobp.2004.10.011

[11] Smitsmans MH, de Bois J, Sonke JJ, Betgen A, Zijp LJ, et al (2005) Automatic prostate localization on cone-beam CT scans for high precision image-guided radiotherapy. Int. J. Radiat. Oncol., Biol., Phys. 63:975–984. 16253772 10.1016/j.ijrobp.2005.07.973

[12] Zhang Y, Zhang L, Zhu XR, Lee AK, Chambers M, et al (2007) Reducing metal artifacts in cone-beam CT images by preprocessing projection data. Int. J. Radiat. Oncol., Biol., Phys. 67:924–932. 17161556 10.1016/j.ijrobp.2006.09.045

[13] Balter JM, Ten Haken RK, Lawrence TS, Lam KL, Robertson JM (1996) Uncertainties in CT-based radiation therapy treatment planning associated with patient breathing. Int. J. Radiat. Oncol., Biol., Phys. 36:167–174. 8823272 10.1016/s0360-3016(96)00275-1

[14] Lujan AE, Balter JM, Ten Haken RK (2003) A method for incorporating organ motion due to breathing into 3D dose calculations in the liver: sensitivity to variations in motion. Med. Phys. 30:2643–2649. 14596301 10.1118/1.1609057

[15] Shimizu S, Shirato H, Kagei K, Nishioka T, Bo X, et al (2000) Impact of respiratory movement on the computed tomographic images of small lung tumors in three-dimensional (3D) radiotherapy. Int. J. Radiat. Oncol., Biol., Phys. 46:1127–1133. 10725622 10.1016/s0360-3016(99)00352-1

[16] Bissonnette JP, Franks KN, Purdie TG, Moseley DJ, Sonke JJ, et al**.** (2009) Quantifying Interfraction and Intrafraction Tumor Motion in Lung Stereotactic Body Radiotherapy Using Respiration-Correlated Cone Beam Computed Tomography. Int. J. Radiat. Oncol., Biol., Phys. 10.1016/j.ijrobp.2008.11.066 19395200

[17] Dietrich L, Jetter S, Tucking T, Nill S, Oelfke U (2006) Linac-integrated 4D cone beam CT: first experimental results. Phys. Med. Biol. 51:2939–2952. 16723776 10.1088/0031-9155/51/11/017

[18] Li T, Koong A, Xing L (2007) Enhanced 4D cone-beam CT with inter-phase motion model. PMed. hys. 34:3688–3695. 10.1118/1.2767144 17926972

[19] Sonke JJ, Zijp L, Remeijer P, van Herk M (2005) Respiratory correlated cone beam CT. Med. Phys. 32:1176–1186. 15895601 10.1118/1.1869074

[20] Zhang QH, Hu YC, Liu FH, Goodman K, Rosenzweig KE, et al (2010) Correction of motion artifacts in cone-beam CT using a patient-specific respiratory motion model. Med. Phys. 37:2901–2909. 20632601 10.1118/1.3397460 PMC2887907

[21] Dzyubak O, Kincaid R, Hertanto A, Hu YC, Pham H, et al (2014) Evaluation of tumor localization in respiration motion-corrected cone-beam CT: Prospective study in lung. Med. Phys. 41:101918. 25281970 10.1118/1.4896101 PMC4281114

[22] Islam MK, Purdie TG, Norrlinger BD, Alasti H, Moseley DJ, et al (2006) Patient dose from kilovoltage cone beam computed tomography imaging in radiation therapy. Med. Phys. 33:1573–1582. 16872065 10.1118/1.2198169

[23] Park JC, Song B, Kim JS, Park SH, Kim HK, et al (2012) Fast compressed sensing-based CBCT reconstruction using Barzilai-Borwein formulation for application to on-line IGRT. Med. Phys. 39:1207–1217. 22380351 10.1118/1.3679865

[24] Jia X, Tian Z, Lou Y, Sonke JJ, Jiang SB (2012) Four-dimensional cone beam CT reconstruction and enhancement using a temporal nonlocal means method. Med. Phys. 39:5592–5602. 22957625 10.1118/1.4745559 PMC3436920

[25] Park JC, Kim JS, Park SH, Liu Z, Song B, et al (2013) Motion-map constrained image reconstruction (MCIR): application to four-dimensional cone-beam computed tomography. Med. Phys. 40:121710. 24320496 10.1118/1.4829504

[26] Dobbins JT III, McAdams HP, Godfrey DJ, Li CM (2008) Digital tomosynthesis of the chest. J. Thor. Imag. 23:86–92. 10.1097/RTI.0b013e318173e162 18520565

[27] Godfrey DJ, McAdams HP, Dobbins JT (2006) Optimization of the matrix inversion tomosynthesis (MITS) impulse response and modulation transfer function characteristics for chest imaging. Med. Phys. 33:655–667. 16878569 10.1118/1.2170398

[28] Yan H, Ren L, Godfrey DJ, Yin F-F (2007) Accelerating reconstruction of reference digital tomosynthesis using graphics hardware. Med. Phys. 34:3768–3776. 17985622 10.1118/1.2779945

[29] Park JC, Park SH, Kim JS, Han Y, Cho MK, et al (2011) Ultra-fast digital tomosynthesis reconstruction using general-purpose GPU programming for image-guided radiation therapy. Technol. Cancer Res. Treat. 10:295–306. 21728386 10.7785/tcrt.2012.500206

[30] Zhang J, Wu J, Godfrey DJ, Fatunase T, Marks LB, et al (2009) Comparing Digital Tomosynthesis to Cone-Beam Ct for Position Verification in Patients Undergoing Partial Breast Irradiation. Int. J. Radiat. Oncol., Biol., Phys. 73:952–957. 19135316 10.1016/j.ijrobp.2008.10.036 PMC2685874

[31] Zhang J, Wu Q, Godfrey D, Yin F (2007) 3D interfraction position verification for patients undergoing partial breast irradiation: Comparing digital tomosynthesis to cone-beam CT. Med. Phys. 34:2607–2607.

[32] Maurer J, Godfrey D, Wang Z, Yin FF (2008) On-board four-dimensional digital tomosynthesis: first experimental results. Med. Phys. 35:3574–3583. 18777918 10.1118/1.2953561

[33] Wang G, Zhao S, Heuscher D (2002) A knowledge-based cone-beam x-ray CT algorithm for dynamic volumetric cardiac imaging. Med. Phys. 29:1807–1822. 12201428 10.1118/1.1494989

[34] Feldkamp LA, Davis LC, Kress JW (1984) Practical Cone-Beam Algorithm. J. Opt. Soc. Am. A. 1:612–619.

[35] Ren L, Godfrey DJ, Yan H, Wu QJ, Yin FF (2008) Automatic registration between reference and on-board digital tomosynthesis images for positioning verification. Med. Phys. 35:664–672. 18383688 10.1118/1.2831903

[36] Yan H, Ren L, Godfrey DJ, Yin FF (2007) Accelerating reconstruction of reference digital tomosynthesis using graphics hardware. Med. Phys. 34:3768–3776. 17985622 10.1118/1.2779945

[37] Maltz JS, Sprenger F, Fuerst J, Paidi A, Fadler F, et al (2009) Fixed gantry tomosynthesis system for radiation therapy image guidance based on a multiple source x-ray tube with carbon nanotube cathodes. Med. Phys. 36:1624–1636. 19544779 10.1118/1.3110067

[38] Santoro J, Kriminski S, Lovelock DM, Rosenzweig K, Mostafavi H, et al (2010) Evaluation of respiration-correlated digital tomosynthesis in lung. Med. Phys. 37:1237–1245. 20384261 10.1118/1.3312276 PMC2905456

